# Engineering a better light sheet in an axicon‐based system using a flattened Gaussian beam of low order

**DOI:** 10.1002/jbio.202100342

**Published:** 2022-02-25

**Authors:** Saiedeh Saghafi, Klaus Becker, Franco Gori, Massih Foroughipour, Christine Bollwein, Meraaj Foroughipour, Katja Steiger, Wilko Weichert, Hans‐Ulrich Dodt

**Affiliations:** ^1^ Section of Bioelectronics Institut für Festkörperelektronik (FKE) Vienna Austria; ^2^ Section of Bioelectronics, Center for Brain Research Medical University of Vienna Vienna Austria; ^3^ Dipartimento di Ingegneria Roma Tre University Rome Italy; ^4^ Institute of Pathology Technical University of Munich Munich Germany

**Keywords:** flattened Gaussian beam, laser beam shaping, meso‐aspheric optics, static light‐sheet microscopy

## Abstract

Lasers are fundamental tools in research and development. The shape of an incident laser beam directly affects the results, when it propagates through complex structured meso‐aspheric optical elements. In conic‐based systems utilizing elements such as axicons, the impact of secondary lobes is mostly overlooked, although the intensity distributions at the central spot and the side‐lobes directly affect the beam properties. We investigate the interaction of two axicons (160° and 170°) with incident beams approximated by Gaussian, high‐order Flattened‐Gaussian, and low‐order Flattened‐Gaussian functions. We demonstrate that replacing an incident Gaussian beam with a low‐order Flattened‐Gaussian beam reduces the secondary lobes and significantly improves the uniformity of the intensity profile. We practically applied this effect in engineering a conic‐aspheric‐based static light‐sheet microscope producing markedly improved results.
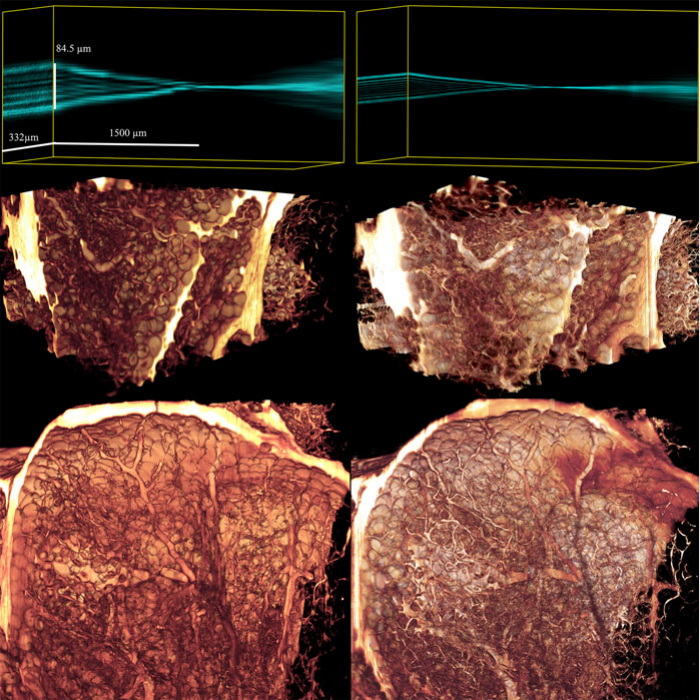

## Graphical abstract

The shape of an incident laser beam directly affects the results when it propagates through complex structured meso‐aspheric optical elements. In conic‐based systems utilizing elements such as axicons, the impact of secondary lobes is mostly overlooked. We investigate the interaction of two axicons (160° and 170°) with incident beams approximated by Gaussian, high‐order Flattened‐Gaussian, and low‐order Flattened‐Gaussian functions. Replacing an incident Gaussian beam with a low‐order Flattened‐Gaussian beam reduced the secondary lobes and significantly improved the uniformity of the intensity profile.

## 1 INTRODUCTION

Laser beam shaping allows tailoring the amplitude and phase of a laser output profile to achieve the desired irradiance distributions required for specific applications. Due to spherical aberration focusing light onto a small spot is a difficult task. Aspherical lenses with complex surface structures have become essential in many optical designs to deal with this problem. In many laser applications, an output beam is required that provides a particular transversal structure (ie, uniform beam, annular beam, spiral beam, as well as perfect Gaussian beam) while maintaining a particular form of spatial and temporal localization of photons [1, 2]. Meso‐aspheric optics deals with the optical properties of conical wavefields and offers ways for building conical surfaces with high precision [3, 4]. An axicon is the most efficient conical optical element for realizing diffraction‐less beams with nondiverging/self‐healing characteristics [5, 6, 7, 8]. An axicon transforms a point light source into a line of focused points that generate a long region with an approximately constant intensity distribution. This distribution tends to become a ring across the propagation axis [5, 6]. The length of the line of focused points depends on the wedge angle of the axicon, the diameter of the incoming beam, and the refractive index of the material. This transformation is homomorphic, thus it is not an imaging element. However, the axicon can be considered as *a beam‐shaping optical element* [3, 5]. It can reshape an incident laser beam with a Gaussian intensity profile into a diffraction‐less beam that can be approximated by a zeroth‐order of the Bessel function [5, 6]. The resulting beam is then defined as an approximation of a Bessel function of the first kind. Depending on the applications, many forms of Axicons (ie, rings, cylinder, conus Axicon, torus Axicon, and even stretched conus axicon) have been developed for generating highly localized beams [9, 10]. Combining an axicon with other optical elements of a particular structure provides a vast degree of freedom for realizing a required laser beam shape, such as uniformly illuminated sheets of light or ring‐shaped beams exhibiting Gaussian‐, Flattened‐Gaussian‐Beam (FGB), and super‐Gaussian irradiance distribution profiles [11, 12, 13, 14]. Light‐sheet has become a scientific rigorous technique for 3D‐imaging microscopy. Over decades, we have been witnessing the continuing developments in light‐sheet microscopy imaging techniques (eg, Ultramicroscopy [15], Selective plane illumination microscopy (SPIM) [16, 17, 18], multi‐directional SPIM (mSPIM), digitally scanned light‐sheet microscopy (DSLM) [19, 20, 21], and Bessel plane illumination microscopy [22, 23], providing 3D images of spectacular quality revealing fine details with high resolution. The improvement of the technique in combination with the development of a plethora of new clearing methods to provide samples with improved transparency and fluorescence preservation enables researchers to achieve cellular and subcellular resolution.

Meso‐aspheric elements, solely or in combination with other optical elements, have been used as an essential part in many optical devices for reshaping multimodes beams into constructed Gaussian beams [24, 25, 26]. Due to the optical characteristics of axicons, they have been used as an essential element in scanned Bessel beam light‐sheet microscopy [21, 27, 28, 29, 30, 31, 32], despite the existence of rings and nonuniform intensity distributions. Many efforts have been made to overcome limitations and unwanted effects that we encounter using conical elements such as Powell lenses or axicon [33, 34, 35, 36]. In most scanning systems, the part of light sheets that is sufficiently thin in the lateral directions is scanned through the sample. Afterwards, the recorded images are stitched together forming a larger image with better quality. In static light‐sheet microscopy (SLSM), we deal with much larger samples (eg, whole mouse brain, cm‐size cancerous tissues as well as the human brain) than scanning light‐sheet microscopy. Due to the impact of secondary lobes, conic‐based systems utilizing optical elements such as axicons are not common in SLSM. Particularly, if the light sheet is as thin as ~2 μm, the interval between two sequentially recorded images can be as small as 2 μm in the z‐direction (detection‐axis). For recording a sample of 5 to 8 mm thickness, we, therefore, would end up with very large image stacks (ie, 2500‐4000). However, sectioning the beam would require laborious work for stitching and postprocessing. This approach when we use several large samples per day is not practical.

Generally, the value of a scientific technique is determined by its contribution to minimizing problems that experimenters encounter. Considering the rapid extension of light‐sheet‐based imaging technique from biological research and medical diagnosis toward industrial applications, methods improving the optical characteristics of the light sheet has been a crucial task.

In this article, we utilize the combination of refractive optics (through lenses and Meso‐aspheric elements) and diffractive optics (with gradual propagation from near‐field to far‐field plane or vice versa) to obtain a light sheet with optimum optical characteristics. We have engineered a self‐design phase/amplitude modulator converting the incident laser beam with Gaussian distribution into a flattened‐Gaussian beam of low‐order, in combination with a patent‐pending design to generate a thin sheet of light with extended uniformity along all axes. According to the Fourier transform, the incident laser beam with a top‐hat or sharp‐edge flat‐top distribution in near‐field belongs to the family of the Sinc function in the far‐field creating a sharp peak with many side lobes and a sharp transition from complete uniform spatial irradiance distribution to distribution containing points with zero intensity. Furthermore, when a focusing unit is illuminated by top‐hat/ sharp‐edge‐flat‐top beam, it has the draw‐back of strong distortion in the intensity distribution in a short distance in either side of the focus along the propagation axis compared with the focusing unit when illuminated by beams with Gaussian distribution. In applications such as static light‐sheet microscopy that a long working distance is required, extending the depth of focus is necessary. Therefore, we have used a flattened‐Gaussian beam of low‐order generated by an amplitude/phase modulator as a possible solution that despite having a semi‐uniform intensity distribution has round edges and possesses a continuous soft transition in intensity from near‐field to constructed far‐field at the focus, forming a distribution approximated with a sharp‐peak and minimum numbers of side‐lobes (improving *M* ^2^). This beam is guided through a unit containing a combination of aspheric lenses, an axicon lens, a soft aperture, and aspheric cylindrical elements to redistribute the irradiance and phase of the beam in a way to generate a thin sheet of light.

It enables us to generate high‐resolution images without sectioning the beam. This design, aside from light‐sheet microscopy, can also be used in many industrial applications (eg, material processing).

We start with a general methodology of describing various laser beam profiles. Then the mutual effects of the conical structures of axicons and the incident beam profiles on the output beam irradiance distributions are analyzed and investigated. The results would be utilized in engineering a patent‐pending design employing conic structure elements for generating a thin sheet of light, which—aside from light‐sheet microscopy—can also be used in many industrial applications (eg, material processing, micromachining, oceanography, environmental science, telecommunications, defense, tissue engineering, and genetic manipulation of microorganisms) [10, 27, 37, 38].

## 2 MATERIALS AND METHODS

### 2.1 General methodology and analytical approach

The field of structured light, amplitude, and phase has been the subject of interest since early studies on laser beam characterization. Bypassing years, the structured laser beam has become a pivotal tool in many applications.

A laser beam accommodating the maximum possible energy in a given region of space has been the subject of interest for many medical and industrial applications. Its bell‐shaped distribution can mathematically be approximated by a Gaussian function that is depicted with the scalar wave equation. Meanwhile, *state‐of‐the‐art* beam‐shaping methods enable users to overcome many of the limitations that we encounter by remaining in the Gaussian regime [2, 36, 39, 40, 41, 42]. Meso‐aspheric elements such as Powell lenses, axicons, and multi‐cylindrical‐conic lenses can convert an approximately Gaussian beam into, for example, a diffraction‐limited Bessel beam/Mathieu beam, Mathieu‐Gauss beam, extended homogeneous line beam, or a light‐sheet of controlled expansion. These advances have opened a new horizon for possible applications of meso‐aspheric elements in beam shaping techniques [24].

A laser beam distribution approximated by the fundamental mode at an arbitrary point along the propagation axis using the angular spectrum method is described by Equation (1):

(1) $$\begin{array}{rcl} \psi \left(r,z\right) & = & \psi_{0}\frac{w_{0}}{w\left(z\right)}e^{\frac{-r^{2}}{w{\left(z\right)}^{2}}}e^{i\left(\mathrm{kz}-\eta \left(z\right)+\frac{\pi r^{2}}{\mathrm{\lambda R}\left(z\right)}\right)}\\ & = & \psi_{0}e^{\left(\frac{-r^{2}}{w_{0}^{2}+{\left(\frac{\mathrm{\lambda z}}{\pi w_{0}}\right)}^{2}}\right)}e^{i\left(\frac{2\mathrm{\pi z}}{\lambda }-\arctan\left(\frac{\mathrm{z\lambda}}{\pi w_{0}^{2}}\right)\right)}\\ & & \times e^{i\left(\frac{\frac{\mathrm{\lambda z}}{\pi }r^{2}}{w_{0}^{4}+{\left(\frac{\mathrm{\lambda z}}{\pi }\right)}^{2}}\right)} \end{array}$$

where *w*_*0*_, *λ*, and *z* is the beam waist, wavelength, and direction of propagation, respectively.

The phase correction ($\eta \left(z\right)$), wavefront radius curvature (*R*(*z*)), and amplitude of the transverse profile ($e^{\frac{-r^{2}}{w{\left(z\right)}^{2}}})$, are given as $\eta \left(z\right)=\arctan\left(\frac{\mathrm{z\lambda}}{\pi w_{0}^{2}}\right)$, $R\left(z\right)=z\left(1+\frac{\pi^{2}w_{0}^{4}}{\lambda^{2}z^{2}}\right)$, and $e^{\left(\frac{-r^{2}}{w_{0}^{2}+{\left(\frac{\mathrm{\lambda z}}{\pi w_{0}}\right)}^{2}}\right)}$, respectively.

At *z* = 0, Equation (1) takes the simple form of $\psi \left(r,z=0\right)=\psi_{0}e^{\left(\frac{-r^{2}}{w_{0}^{2}}\right)}$. To determine any irradiance distribution at any arbitrary point within an optical system the optical field must be characterized throughout the system. This field can be, for example, considered as a bundle of rays along the z‐axis with a dedicated relationship between their amplitude and phase.

**FIGURE 1:**
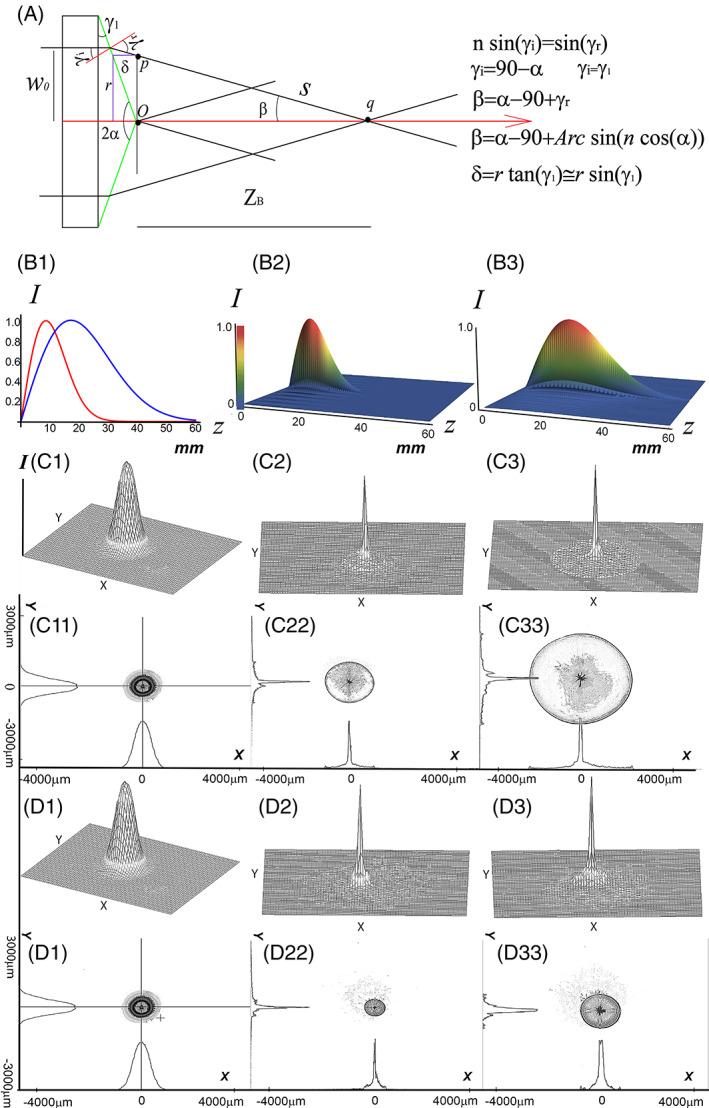
Axial and transversal intensity profiles of a fundamental laser beam striking an axicon lens. (A) Beam propagation in a well‐structured Axicon, (B1) Simulated 2D axial intensity distribution for two Axicon of 160°and 170° if they are illuminated by a laser beam (1.5 mm spot‐size/ 532 nm/*TEM*_00_), (B2) 3D normalized axial intensity profile of the Bessel beam formed by a 160°‐axicon (including its Fresnel zone) along 60 mm distance from the tip of the axicon, (B3) 3D normalized axial intensity profile of the Bessel beam formed by a 170°‐axicon (including its Fresnel zone) along 60 mm distance from the tip of the axicon, (C1) Three‐dimensional measured intensity profile of the incident laser beam striking the tip of 160°‐axicon (position‐1/z = 0), (C11) The contour plot of a measured intensity profile of the incident laser beam striking the tip of 160°‐axicon (position‐1/z = 0), (C2) Three‐dimensional measured intensity profile of the incident beam striking a 160°‐axicon at the position where the axial intensity reaches its maximum value (position‐2/z = 9.2 mm), (C22) The contour plot of the measured intensity profile of the incident beam striking a 160°‐axicon at the position where the axial intensity reaches its maximum value (position‐2/z = 9.2 mm), (C3) Three‐dimensional measured intensity profile of the incident beam striking a 160°‐axicon at the position where the axial intensity drops by 86% of its maximum value (position‐3/z = 22.3 mm), (C33) The contour plot of the measured intensity profile of the incident beam striking a 160°‐axicon at the position where the axial intensity drops by 86% of its maximum value (position‐3/22.3 mm), D1, D11, D2, D22, D3, and D33 have the same description as C1, C11, C2, C22, C3, and C33, respectively, just the axicon is replaced by a 170°‐axicon, when Position‐1, Position‐2, and Position‐3 are located approximately at *z* = 0, *z* = 17.7 mm, and 45.7 mm, respectively

Therefore, the formed irradiance distribution can be predicted at any arbitrary point by superpositioning these rays, considering their amplitude and phase transmittance. If a radially symmetric Gaussian field given by Equation (1) is considered as a bundle of parallel rays along the z‐axis and if this incident Gaussian beam strikes upon a convergent Meso‐aspheric conical element such as an axicon with a refractive index *n* (Figure 1A), the rays would refract individually upon arrival at the conical surface.

Thus, a complex amplitude and phase transmittance would occur if the amplitude transmittance associated with frequency is $\varphi_{A}\left(\omega \right)$ and the phase transmittance is $\varphi_{\phi }\left(r\right)=e^{i\frac{2\pi }{\lambda }r\sin\beta }$, where β is the axicon deflection angle. Using trigonometry and Snell's law, β is related to the apex angle of the axicon through Equation (2) as can be seen in Figure 1A;

(2) $$\beta =\alpha -\frac{\pi }{2}+\mathrm{Arc}\;\sin\left(n\;\cos\alpha \right).$$

Therefore, the total complex transmittance is given by the product of $\varphi_{A}\left(\omega \right)$ and $\varphi_{\phi }\left(r\right)$ as $\varphi_{\mathrm{A\phi}}\left(r,\omega \right)=\varphi_{A}\left(\omega \right)\cdot \varphi_{\phi }\left(r\right)$. The optical path difference of the field at point *p* compared with the field at point *o*, as shown in Figure 1A, is given by Equation (3):

(3) $$∆=\frac{2\pi }{\lambda }\delta \left(\;1-n\right)≅\frac{2\pi }{\lambda }\;r\;\gamma_{1}\left(\;1-n\right),$$

where $\gamma_{1}=180-2\alpha$ is the side angle/Physical angle of the meso‐aspheric cone of the axicon. The length of Bessel zone/Depth of Focus (DOF) is given by $Z_{B}=\frac{w}{\tan\beta }$ and is determined by the fabrication quality of the cone structure of the Axicon, the incident beam width, the radius of the axicon front face, the refractive index of an axicon, and the refracted angle.

Using the general form for Huygens‐Fresnel diffraction integral [43] in combination with the field described by Equation (1) and the complex transmittance of the axicon lens we can evaluate the gradual change in the incident Gaussian field after passing through the axicon as given by Equation (4):

(4) $$U\left(r,\omega \right)=A\int_{s}\psi \left(r,z=0\right)\varphi_{\mathrm{A\phi}}\left(r,\omega \right)\frac{e^{\mathrm{iks}}}{s}\mathrm{dr}=\;\frac{\mathrm{Ai}}{\lambda }\int_{s}\psi_{o}e^{\left(\frac{-r^{2}}{w_{0}^{2}}\right)}\varphi_{A}\left(\omega \right)e^{\frac{i2\mathrm{\pi r}}{\lambda }\gamma_{1}\left(1-n\right)}\frac{e^{i\frac{2\mathrm{\pi s}}{\lambda }}}{s}\mathrm{dr}$$

where *s* is the distance between points *p* and *q* as shown in Figure 1A.

Using Equation (4), the axial field intensity distribution along DOF of a rotationally symmetric incident Gaussian beam can be approximated for certain values and distances by the product of the zero‐order Bessel function and a Gaussian distribution given by Equation (5):

(5) $$I_{\mathrm{DOF}}={\left|U\left(r,z\right)\right|}^{2}≅A\frac{{\left(2\pi \right)}^{2}}{\lambda }\gamma_{1}^{2}{\left(n-1\right)}^{2}ze^{\frac{-2\gamma_{1}^{2}{\left(n-1\right)}^{2}z^{2}}{w_{0}^{2}}}{\left|J_{0}\left(\frac{2\pi \left(n-1\right)r\gamma_{1}}{\lambda }\right)\right|}^{2}$$

where *A* is the normalizing factor. Other parameters such as $\gamma_{1}$, *w*_*0*_, *λ*, and *z* have the same meaning as described before.

The normalized axial intensity profiles along the Fresnel zone are simulated and compared for two well‐structured axicons having an apex angle of 160° and 170° (Figure 1B1).

The individual 3D distribution of the axial intensity profiles of an incident Gaussian beam (532 nm/~1.5 mm‐*TEM*_00_ mode/Edmund Optics) striking axicons of 160° and 170° are plotted in Figure 1B2 and B3, respectively.

As obvious, the intensity profile of the Bessel beam generated within the depth of focus (DOF) has no uniform intensity distribution along the propagation axis independently of the value of the apex angle. Furthermore, the transversal intensity profiles of the laser beams impinged on these two axicons are measured and plotted at three positions within the Fresnel zone.

The contour plot and their 3D‐distributions are measured using a LaserCam‐HRTM (Coherent Inc./Germany) at the tip of two axicons and at two positions along the propagation axis, where the related axial intensity reaches its maximum value and when it drops by 86% of the maximum value as demonstrated in Figures.1C(1‐3), C(11‐33), D(1‐3), and D(11‐33).

The sensor element of the CCD of LaserCam‐HRTM (Coherent Inc./Germany) has 1280*1024 pixels. The size of each pixel is 6.5 μm*6.5 μm providing an 8320 μm*6656 μm capturing area as shown in Figure 1C1 to D33. This CCD camera can capture continuous (CW) as well as pulsed modes.

**FIGURE 2:**
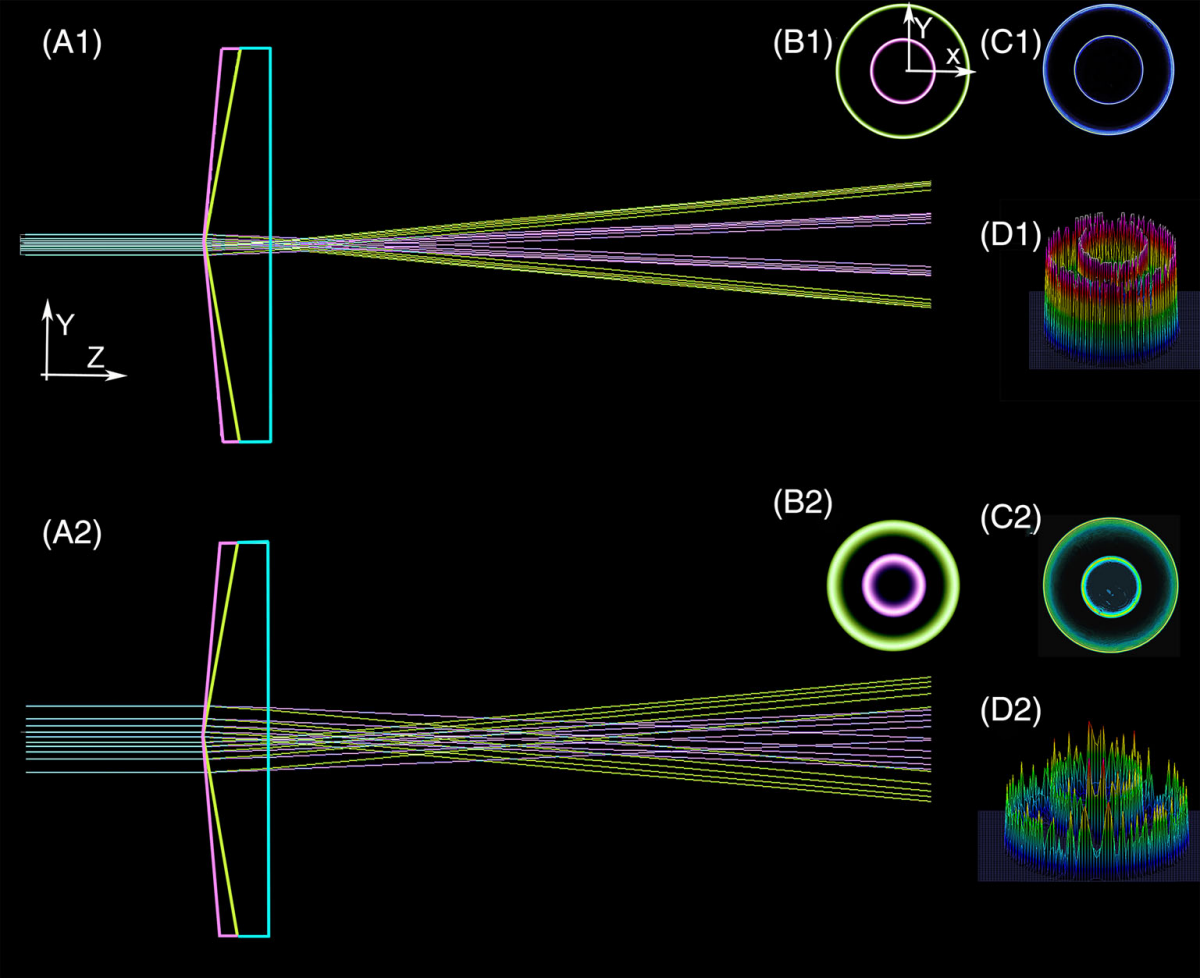
Association between the width of incident Gaussian beams and axicon lenses of different apex angles, where *Z* is the propagation axis while X and Y are position coordinates at the cross‐section of the beam. It shows the formation of the ring‐shaped beam distribution through propagation. (A1) Using Zemax (Optostudio, USA), the propagation of a fundamental mode laser beam (1.5 mm/532 nm) that is incident upon two axicons of 170° (Conic factor: −131.646—Magenta color) and 160° (conic factor: −33.1634—Green color) is shown. The blue color represents the parts that are common between the two axicons. (B1) Simulated 2D‐beam intensity profile at the X‐Y plane normal to the propagation axis 45 mm away from the tip of the axicon (The green and magenta colors are related to the axicons of 160° and 170°, respectively), (C1) The measured 2D beam intensity profile obtained by LaserCam‐HRTM (Coherent/Germany) 45 mm away from the tip of the axicon (The green and magenta colors are related to the axicon of 160° and 170° (Asphericon/Germany), respectively), (D1) The 3D‐measured beam profiles obtained by LaserCam‐HRTM (Coherent/Germany) 45 mm away from the tip of the two axicons (The inner distribution is related to 170° axicon while the outer distribution representing larger expansion is related to 160° axicon, A2) Showing the propagation of a 5 mm laser beam (532 nm) with Gaussian intensity distribution through two axicons of 170° (conic factor: −131.646—Magenta color) and 160° (conic factor: −33.1634—Green color) using Zemax software (Optostudio/USA); B2, C2, and, D2) They are demonstrating the effects as described at B1, C1, and D1 for a 5 mm incident Gaussian beam, respectively

The refraction of the beam that is propagating through the axicon will differ depending on the size of the incident beam, its intensity profile, the apex angle or the physical angle of the axicon lens, and the refractive index of the medium. The simulated and measured data for two axicons (Apex angle:160° and 170°, Asphericon, Germany) for a 1.5 mm and a 5 mm laser beam (Sapphire laser/532 nm/200mw/Coherent Inc./Germany) is shown in Figure 2.

It can be easily seen that the smaller the apex angle is the shorter the length of the focused points, and the faster the divergence angle becomes. Furthermore, looking at the cross‐section of the beam (X‐Y plane), it is obvious that the refracted Gaussian distribution has the intendancy of becoming denser toward the outer area in comparison with the incident Gaussian distribution (which is denser at the center).

### 2.2 Alteration of Gaussian beam, FGB of a high order, and FGB of low order profiles through propagation

#### 2.2.1 In air

Here, we investigate the effects of the conic structure of an axicon on the alteration of the incident beam intensity profile. We considered beams that can be described by Gaussian function (fundamental mode) as well as beams with a uniform intensity distribution that can be approximated by the model given by Gori in 1996 [12]. Based on the finite superposition of the Laguerre‐Gaussian function, the so‐called “Flattened Gaussian beam‐FGB” was developed and takes the form of Equation (6) [12]:

(6) $$\psi_{N}\left(r,z\right)\approx \frac{w_{N}\left(0\right)}{w_{N}\left(z\right)}e^{\left(i\left(\mathrm{kz}-\Phi_{N}\left(z\right)+\frac{k}{2R_{N}\left(z\right)}\right)\right)}e^{\left(\frac{-r^{2}}{w_{N}^{2}\left(z\right)}\right)}\times \;\;\;\;\;\;\;\;\;\;\;\;\;\;\sum_{n=0}^{N}C_{n}^{N}L_{n}\left[\frac{2r^{2}}{w_{N}^{2}\left(z\right)}\right]e^{\left(-2i{\mathrm{n\Phi}}_{N}\left(z\right)\right)}.$$

where $C_{n}^{N}$ is related to a binomial coefficient and is given by $C_{n}^{N}={\left(-1\right)}^{n}\sum_{m=n}^{N}\frac{\left(\begin{array}{c} m\\ n \end{array}\right)}{2^{m}}$ controlling the superposition of Laguerre polynomials.

Wavenumber, the beam spot size at an arbitrary point along the propagation axis, the radius of the curvature, and the phase shift are given by *k*, $w_{N}\left(z\right)$, $R_{N}\left(z\right)$, and $\Phi_{N}\left(z\right)$, respectively.

They are formulated as $k=\frac{2\pi }{\lambda }$, $w_{N}\left(z\right)=w_{N}\left(0\right)\sqrt{1+{\left(\frac{\mathrm{\lambda z}}{\pi w_{N}^{2}\left(0\right)}\right)}^{2}}$, $R_{N}\left(z\right)=z\left(1+{\left(\frac{\pi w_{N}^{2}\left(0\right)}{\mathrm{\lambda z}}\right)}^{2}\right)$, $\Phi_{N}\left(z\right)=\arctan\left[\frac{\mathrm{\lambda z}}{\pi w_{N}^{2}\left(0\right)}\right].$

In addition, $w_{N}\left(0\right)$ is the spot size at *z* = 0, which is related to the beam waist of the FGB, $w_{0}$, through $w_{N}\left(0\right)=\frac{w_{0}}{\sqrt{N}}$.

Equation 6 provides a valid approximation for the beam with the fundamental mode distribution (Gaussian beam) as well as for beams having top‐hat intensity profiles with different degrees of distribution's flatness and shoulder's steepness.

It is known that the beam shape and diameter alter while propagating from the near‐field toward the far‐field along the propagation axis. This alteration speeds up significantly if the beam is incident upon a convergent optical element such as a lens (or a converging axicon).

**FIGURE 3:**
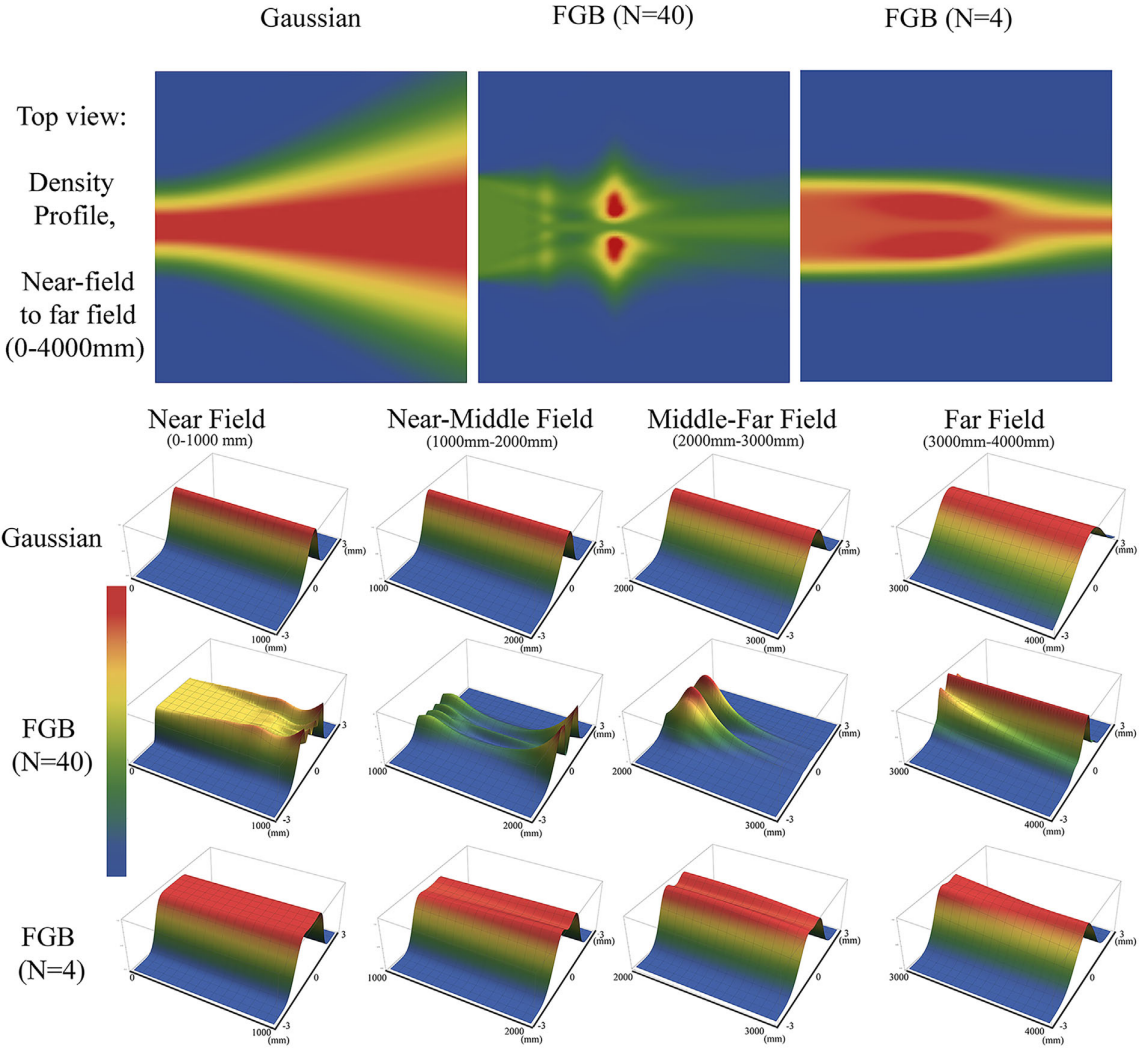
First raw: The top view of the simulated density profile for three 488 nm laser beams described by Gaussian function, high‐order FGB (*N* = 40), and low order FGB (*N* = 4) of the same beam width (~1.5 mm) when encircling 86% of energy from near‐ to the far‐field (~ 0‐4000 mm). The 3D profiles of subsequent evolution in intensity distribution at the near‐, Near/Middle‐, Middle/Far‐, and the Far‐field when the incident beam is approximated by a Gaussian, high‐order FGB (*N* = 40), and the low‐order FGB (*N* = 4) are plotted in the second, third, and fourth raw, respectively

Increasing the beam order (*N*) or decreasing the size of the incident beam width accelerates this process. Figure 3 demonstrates the alteration of the intensity profiles of the beam described by Equation 6 for the beam order of *N* = 1, *N* = 4, and *N* = 40 along the propagation axis (*z*) in Air. The propagation distance is divided into four regions: the near‐field, near/middle‐field, middle/far‐field, and the far‐field.

As we can see from Figure 3 a Gaussian beam remains Gaussian while it is propagating in free space. Afterward, the near‐field radius and waist increase accordingly with the distance. The intensity profile of the high‐order FGB (*N* = 40) in free space goes through a complex alteration from near‐field to far‐field, making it a problematic candidate for many practical applications, even though it may look very interesting in the near‐field.

In contrast, the FGB of low order (*N* = 4) preserves the energy along the propagation axis in free space. This, as well as a uniform intensity profile that is extended beyond the near‐field, is required by many applications. The preservation of shape, width, and energy becomes important when the beam propagates through optical systems.

#### 2.2.2 Passing through conic elements

It is known that if an appropriate scaling factor is used, the far‐field distribution can be formed much closer to the illumination source by using a focusing element such as a converging lens or positive axicon [44].

Therefore, when the beam is traveling through converging‐optical elements such as convex lenses, the alteration in optical characteristics of the beam that usually occurs in a relatively long distance between the near‐field (immediately after the lens) to far‐field (at physical infinity) would occur in a much shorter length between the exiting lens aperture up to the focal point where a virtual far‐field is formed. The irradiance profile can go through significant alteration due to the propagation through a complex optical medium such as meso‐aspheric conic elements (eg, axicon).

In many studies on axicons the impact of the secondary lobes, as well as the nonuniformity of intensity distributions, either at the central spot or at the side lobes, are overlooked [45, 46]. This is due to the rapid decay of side lobes with increasing distance to the optical axis. These properties are directly affected and controlled by the distribution profile of the incident beam.

**FIGURE 4:**
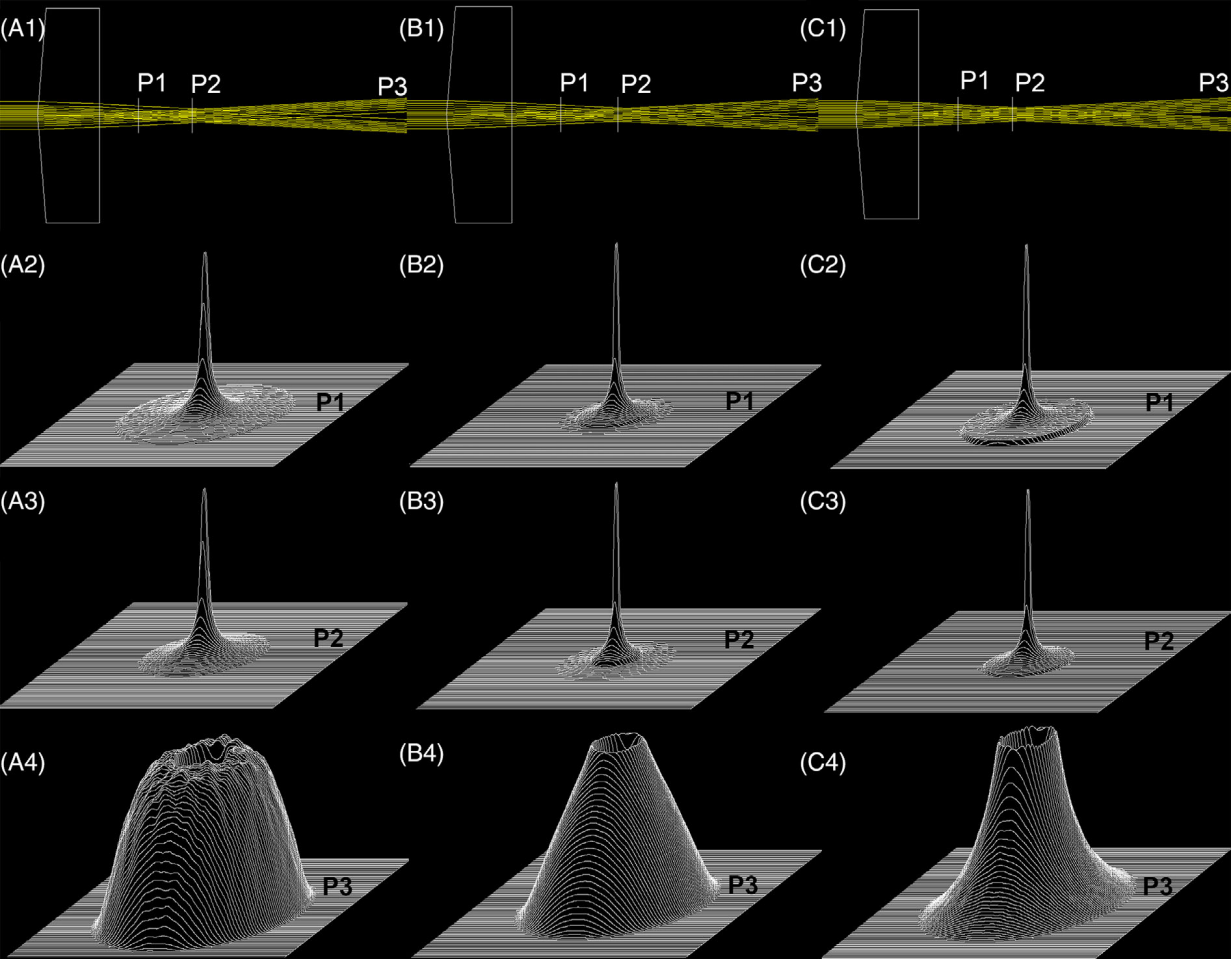
Effects of an Axicon of 170° (conic factor: −131.646) on a Gaussian beam, FGB of order 4 and FGB of order 40 (nearly top‐hat beam). The effects are compared at three positions, P1, P2, and P3. (A1) Top‐view of the Gaussian beam propagation through a well‐structured axicon of 170° along the z‐axis, (A2) Three‐dimensional distribution of transversal irradiance profile along the XY plane at position P1, (A3) Three‐dimensional distribution of transversal irradiance profile along XY plane at position P2 that is located at 10 mm distance from P1, (A4) Three‐dimensional distribution of transversal irradiance profile along the XY plane at position P3 that is located at 50 mm distance from the back of the axicon, (B1‐B4) Figures B1‐B4 have the same description as given in A1‐A4 considering the incident beam is an FGB of low order (*N* = 4), (C1‐C4) Images C1‐C4 have the same description as presented for A1‐A4 considering the incident beam is an FGB of high order (*N* = 40)/Top‐hat beam

Figure 4 demonstrates the effects of an Axicon lens of 170° (Conic factor: −131.646) on three different beam profiles that are approximated by (A) A Gaussian Beam, (B) A FGB of low mode order (*N* = 4), and (C) A top‐hat beam or FGB of high order (*N* = 40) of the same width.

Compared with a Gaussian or a high‐order FGB (*N* = 40), using an FGB of low‐order (*N* = 4) reduces the impact of the secondary side lobes in a Bessel‐like beam generated by an axicon (Figure 4).

Furthermore, preservation of energy and uniformity of the intensity distribution in the central spot is optimized in the low‐order FGB (*N* = 4). This makes it a good candidate for many applications such as axicon‐based static light‐sheet microscopy.

## 3 AXICON‐BASED STATIC LIGHT‐SHEET MICROSCOPY

### 3.1 Design and results

An axicon is commonly used in scanning‐based light‐sheet microscopy due to its self‐healing properties and its robustness against deflection at objects [5, 6]. However, utilizing axicons is not common in static light‐sheet microscopy due to the existence of the side lobes.

We improved the performance of our existing setup for static light‐sheet microscopy in a two steps process. Initially, we converted the incident laser beam with Gaussian distribution to an FGB of low order. In the second step, the optical meso‐aspheric elements of specific structures were accommodated in the light generator system to produce a thin sheet of light with optimized optical characteristics.

As demonstrated by Figure 4 an FGB of low order (*N* = 4) is most suitable for this purpose, since it provides the most uniform intensity distribution with the lowest amount of side shoulders, compared with the other profiles.

#### 3.1.1 Flattened‐Gaussian‐beam‐generator‐unit

**FIGURE 5:**
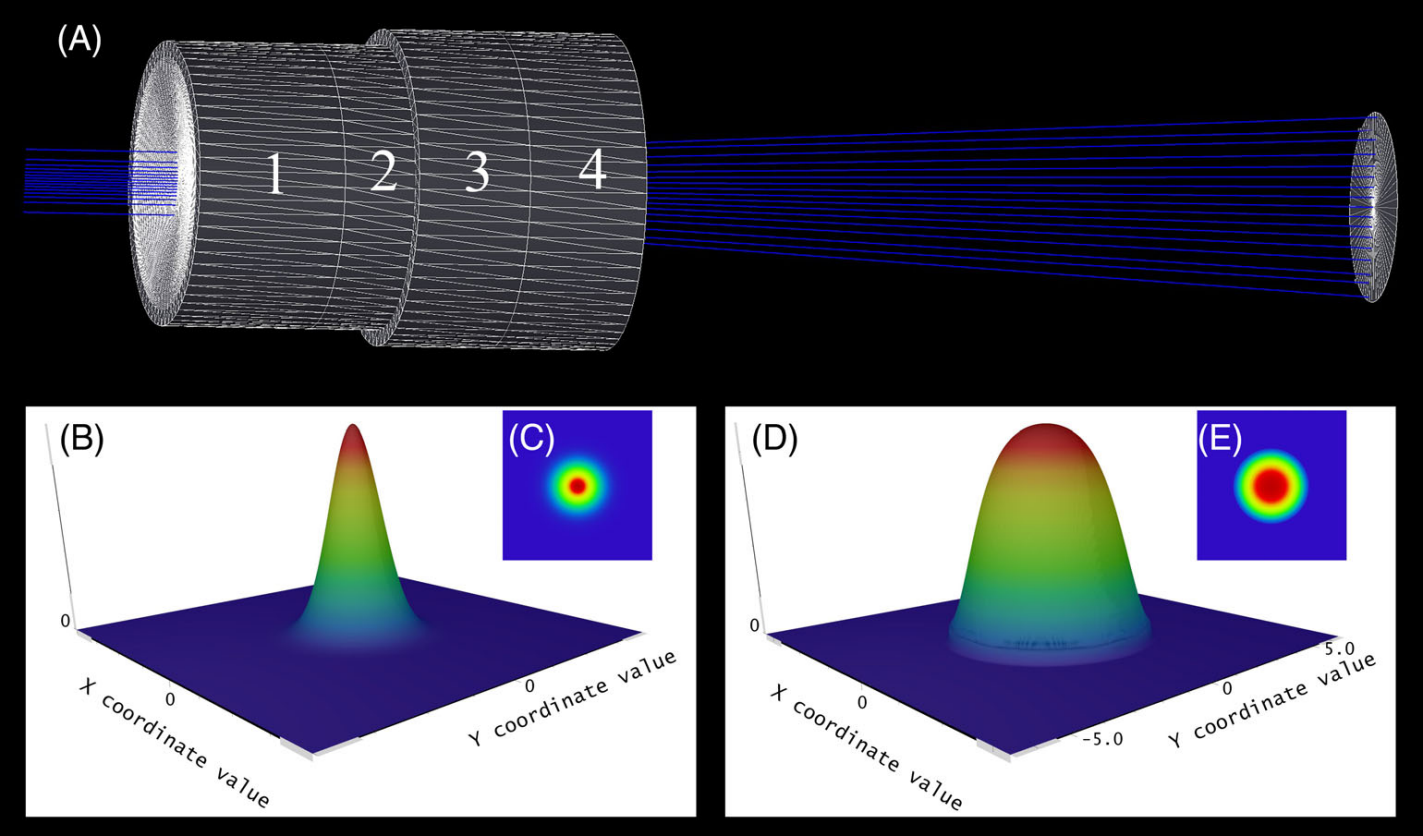
Schematic design of a low‐order flattened gaussian beam generator unit (FGBGU). (A) The schematics of FGBGU containing four optical elements reshaping a Gaussian beam into an FGB of low order (*N* = 4), (B) Three‐dimensional irradiance profile of the incident beam with Gaussian distribution (~1.5 mm beam width/1.2 Gaussian factor/ 488 nm), (C) The contour plot of the transversal irradiance distribution of the incident Gaussian beam, (D) Three‐dimensional irradiance distribution of the generated low‐order FGB (*N* = 4) at 50 mm distance, (E) The contour plot of the transversal irradiance distribution of low‐order FGB (*N* = 4)

The Flattened‐Gaussian‐Beam‐Generator‐Unit (FGBGU) is an amplitude modulator comprising four cemented optical elements as shown in Figure 5. The first one is a conic aspheric surface with a radius of curvature of −7.825 mm that is made from a material of the lanthanoid family (*n* = 1.6591).

This minimizes dispersion effects and optimizes the clarity of the formed images.

To eliminate undesirable intensity variations in the tails of the irradiance distribution, a 2 mm soft aperture (ie, an apodizing filter that its normalized optical density is defined by a polynomial function of order four) is cemented to the flat surface of the first element.

The third cemented part is an aspheric optical element made from Titanate with a radius of curvature of 90.758 mm. To optimize the intensity distribution for achieving an FGB of low order (*N* = 4) the final optical element is a negative conic aspheric lens with a radius of curvature of −15.785 that is made from a material of the lanthanoid family (*n* = 1.8685).

Using FGBGU in combination with an axicon‐based light sheet generator, we can improve the light sheet markedly.

#### 3.1.2 Axicon based light‐sheet unit microscope

By guiding the output beam of the FGBGU toward a 170° axicon (Asphericon/Germany) that is placed at a 50 mm distance, we get a beam that behaves as predicted in Figure 3B1‐B4. These two, combined with another precision‐aspherized‐achromatic lens of focal length $F_{1}$ placed at a distance of $2F_{1}$ from the flat surface of the axico*n*, enable us to obtain a magnified axially symmetric flattened Gaussian beam with optimized parameters. By guiding this beam toward an achromatic‐acylinder lens of focal length $F_{2}$ placed at $F_{1}+F_{2}$, a controlled ellipticity is introduced to the beam structure. Finally, a positive achromatic‐acylinder lens of focal F3 placed at a distance of $\sqrt{2}F_{2}$ from $F_{2}$ enables us to form a very thin sheet of light.

This light sheet is remarkably improved compared with a light‐sheet generated by a slit‐aperture and a cylindrical lens, or a light‐sheet generated by our patented system using a Gaussian beam as input. All the details are given in Supporting information S1.

**FIGURE 6:**
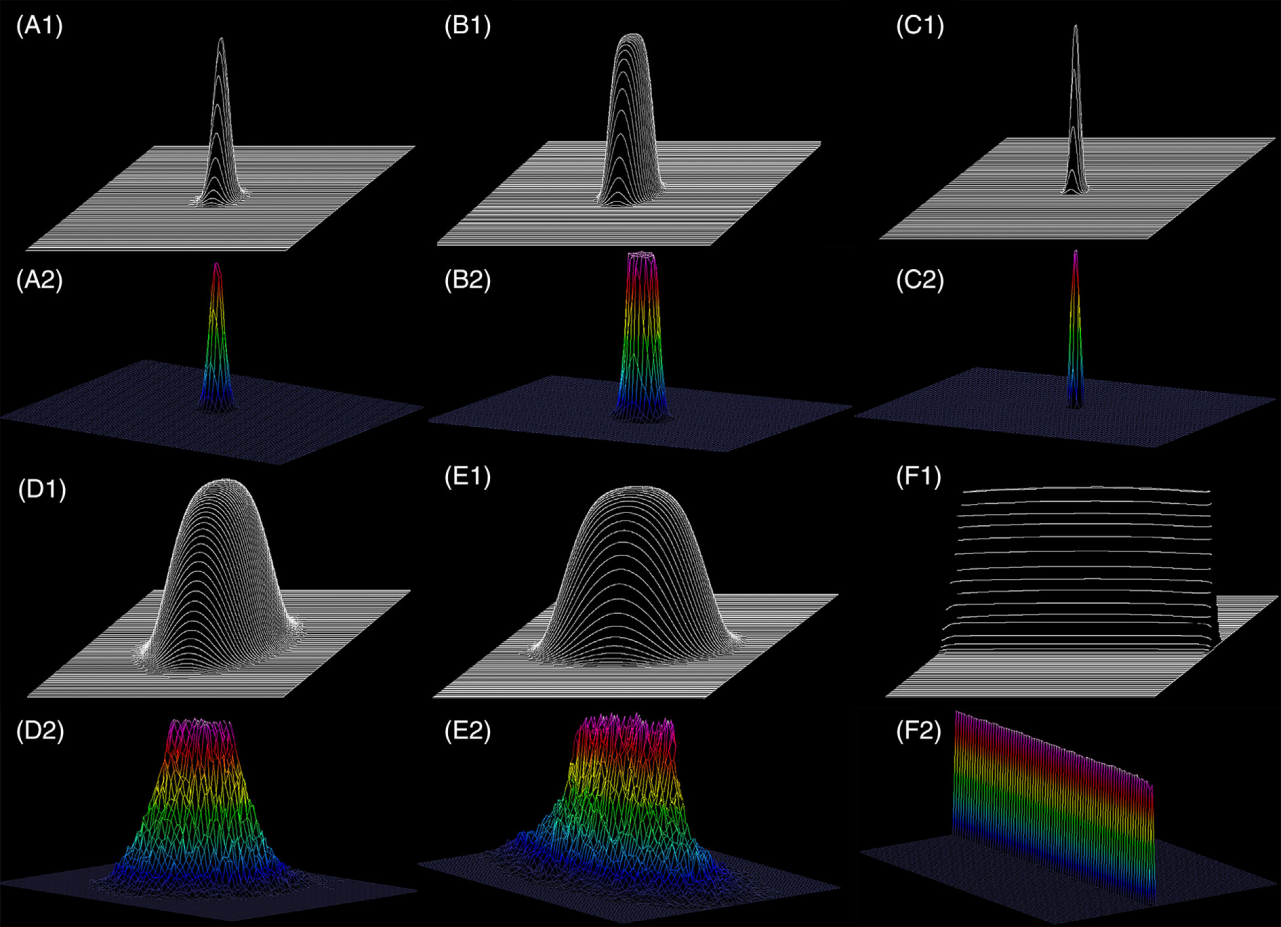
The excellent agreement between simulated and measured data shows the gradual alteration in the intensity profile of a laser beam from Gaussian toward the formation of a thin light sheet. (A1‐A2) Simulated intensity profile (SIP) and measured intensity profile (MIP) of the incident beam (~1 mm width) on the front surface of the first FGBGU generating an FGB of order 4. The intensity profile was measured by a Laser‐Cam‐HRTM beam analyzer (Coherent Inc., Germany). (B1‐B2) SIP and MIP of the FGB (*N* = 4) incident on the front surface of axicon lens (170°/Asphericon/Germany), (C1‐C2) SIP and MIP of the high‐density irradiance with uniform distribution generated by the last two elements. It is incident on the front surface of the third lens producing an optimized axially symmetric FGB with the required width, phase, and uniformity of the irradiance distribution. (D1‐D2) SIP and MIP of the incident beam on the front surface of Lens 4, which is an achromatic cylindrical lens. This lens introduces a controlled ellipticity in the intensity profile. (E1‐E2) SIP and MIP of the generated elliptical incident beam generated by the fourth lens on the front surface of the fifth lens that is another achromatic cylindrical lens. During propagation, the beam further reshapes into an elliptical FGB of the optimized parameter. (F1‐F2) SIP and MIP of the focused light‐sheet that were generated after passing the last lens

Using Zemax‐OpticStudio (USA), the gradual alteration of intensity profiles of the incident Gaussian beam toward the light sheet formation is simulated. A light‐sheet microscopy system was built based on this design and the beam profiles were measured at comparable positions. Figure 6 demonstrates the high degree of agreement.

## 4 RESULTS

**FIGURE 7:**
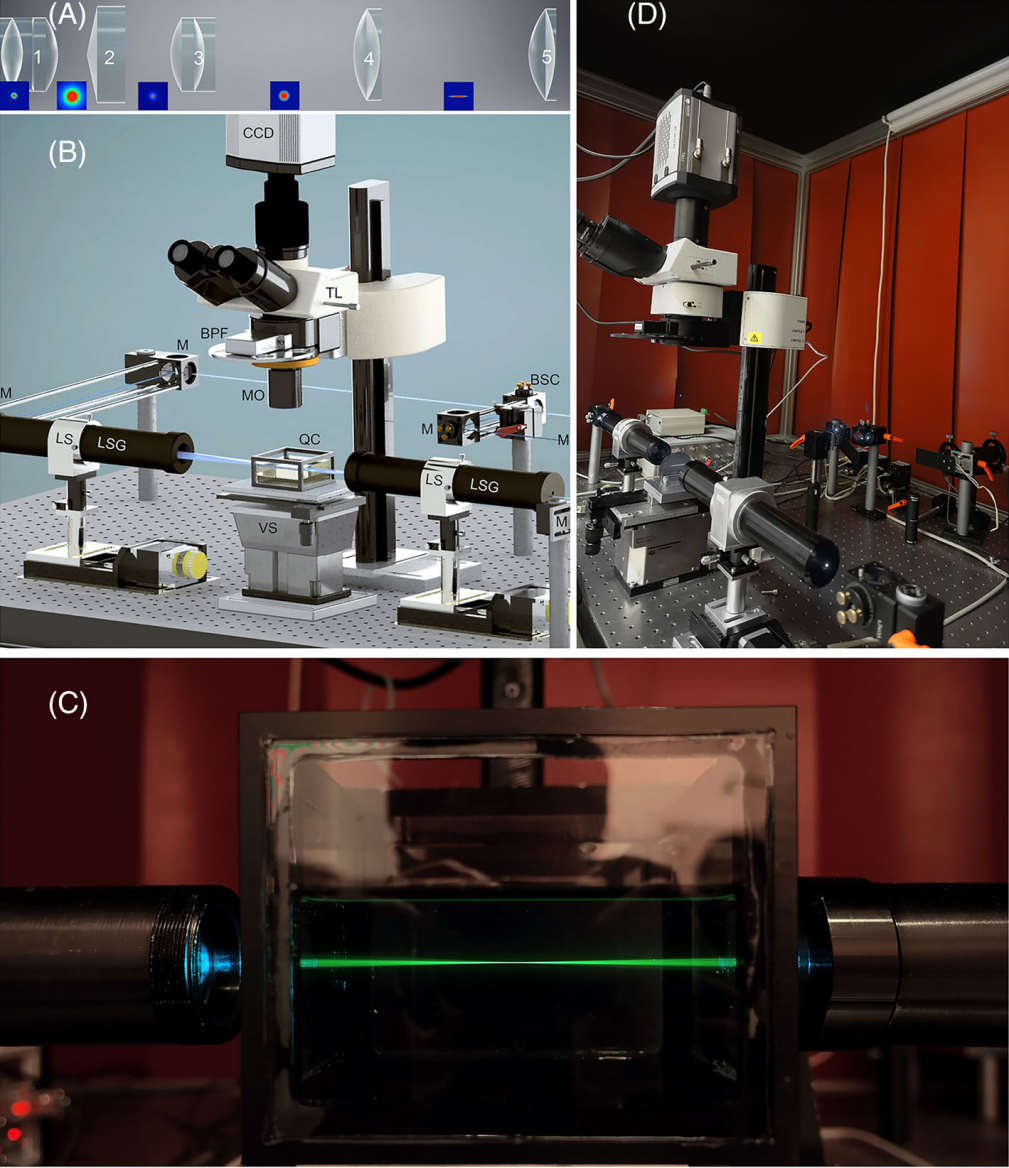
Axicon‐based static light‐sheet microscope. (A) The optical design of the LSG unit. It contains five elements: (1) a precision FGBGU comprising aspherized‐achromatic optical components, (2) An axicon with an apex angle of 170° (Asphericon/Germany), (3) A precision aspherized‐achromatic lens of focal length $F_{1}$ (Edmund Optics, UK), (4) an achromatic‐acylinder lens of focal length $F_{2}$ placed at $F_{1}+F_{2}$ (mm) distance from the third optical element. (5) An achromatic cylindrical lens of focal length $F_{3}$ placed at a distance of $\sqrt{2}F_{2}$ from the fourth lens. (B) The system contains Sapphire laser units for fluorescence excitation, a beam splitter cube (BSC), 45° silver mirrors (M), two light‐sheet generators (LSG) nits, two linear stages (LS) for moving the LSG units along the beam propagation axis (z), a computer‐controlled vertical stage (VS) for moving the sample through the light sheet, and a Quartz container (QC). The detecting unit contains objectives equipped with a modulator (MO) for compensating refractive index mismatch, a Tube lens (TL) equipped with Band Pass Filter (BPF) wheels, and a CCD Camera. (C) The developed axicon‐based light‐sheet microscopy at TU Wien. (D) Photo of the double‐sided light‐sheet along the propagation axis in an 80 mm*50 mm*50 mm container. The container is filled with an aqueous fluorescein solution to visualize the light pathway

A sketch of our setup is presented in Figure 7. It is equipped with a 200 mW Sapphire laser unit (Coherent Inc./Germany) emitting a 488 nm continuous beam with a Gaussian distribution profile for fluorescence excitation. All lenses have an anti‐reflection coating.

The beam is divided into two equal portions by a Beam Splitter Cube‐BSC (Qioptiq/Germany) and fed into two identical light sheet generator (LSG) units by silver elliptical plane mirrors‐M (Qioptiq/Germany). These two units are assembled on two linear stages, LS, to illuminate a transparent sample that is placed in a specimen chamber made from Quartz (Q).

The specimen chamber is filled with a medium of the same refractive index as the sample. It is located on top of a computer‐controlled vertical elevation stage (VS) with an adjustment precision of 100 nm (Es‐100, PI‐Micos GmbH, Germany). The detection unit includes a customized microscope equipped with custom made objectives (MO) for compensating refractive index mismatches, a tube lens (TL), a computer‐controlled band‐pass filter (BPF) wheel for blocking the fluorescence excitation light, and a scientific grade CCD camera (Neo 5.5 sCMOS, 5 Megapixel, 6.5 μm pixel, UK).

This axicon‐based light‐sheet microscope has been applied for imaging chemically cleared human adipose mammary tissue. The utilized clearing method was the standard 3DISCO clearing, which means the dehydration was obtained using an ascending alcohol series and dibenzyl‐ether (DBE) that was used as the clearing solution. Dehydration lasted for 1 month.

**FIGURE 8:**
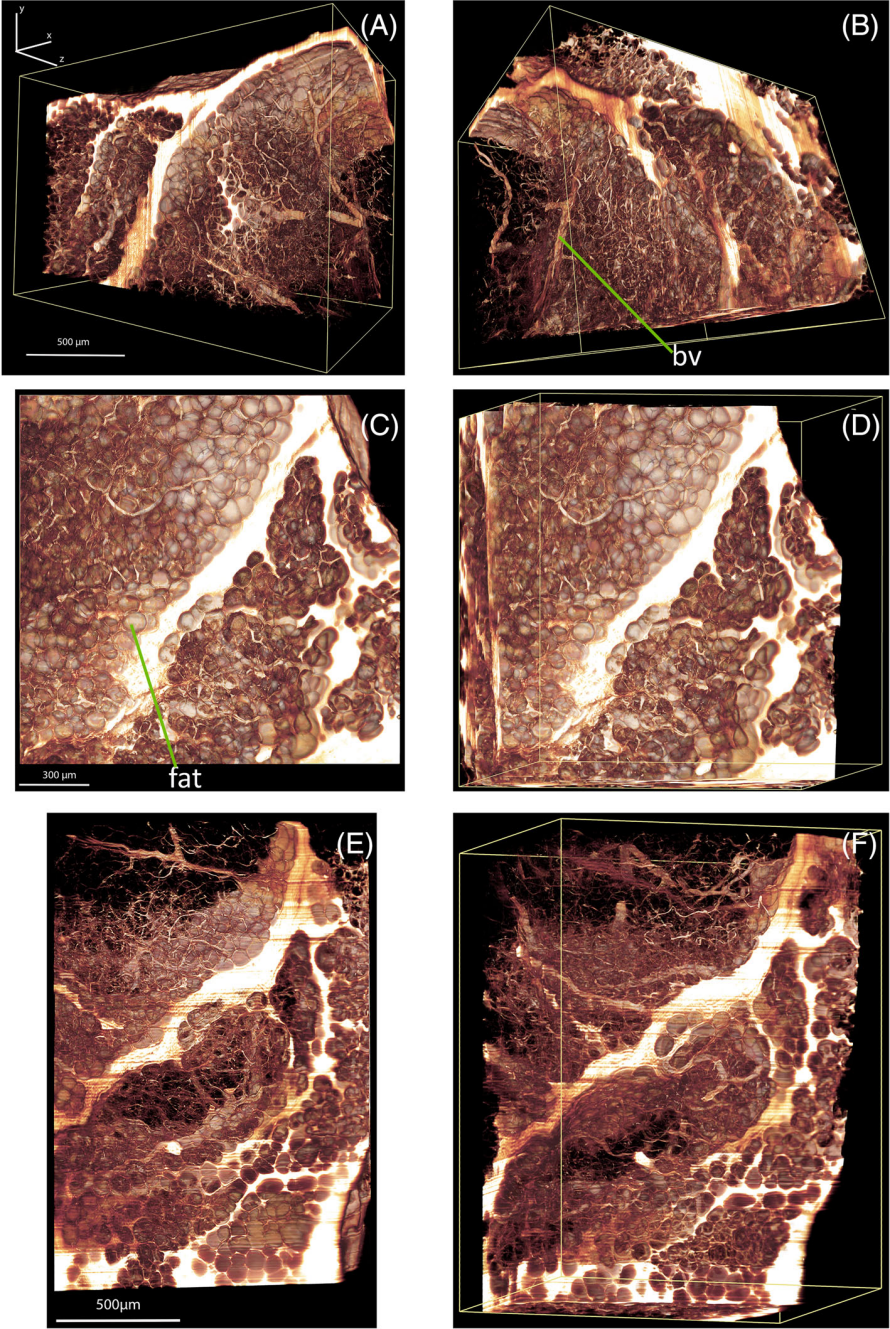
Three‐dimensional reconstructions of unstained, adipose mammary tissue of the human breast were obtained by the Axicon‐based light‐sheet microscopy setup described here. For imaging, a 10X objective (NA 0.3, Olympus/Japan) equipped with a custom‐made modulator for compensating the refractive index mismatch was used. 3D‐reconstructions were generated from 2100 optical sections using Amira (Thermo Fisher Scientific, USA). (A‐F): views of the same sample from different directions. *fat*: fat cells, *bv*: blood vessels. Visualization 1 represents a 3D reconstruction and Visualization 2 demonstrates highly isotropic, high‐resolution 3D views of the human breast tissue. See Visualization 3 for fine details of the tissue structure that were revealed by this noninvasive imaging technique

Using Amira (Thermo Fisher Scientific, USA), 3D‐reconstructions were generated from 2100 optical sections that were obtained by a 10X objective (NA 0.3, Olympus/Japan) equipped with a custom‐made modulator for compensating the refractive index mismatch revealing the fine details of fat cells and some blood vessels filling the lobules between the septa. This example demonstrates that our light sheet allows nearly isotropic imaging of several mm large samples as shown in Figure 8. This should become very important for 3D imaging of cancerous tissue.

In order to use biological material (cells and tissues) for scientific and technical research, several considerations are required. One of the main issues is the ethical aspect.

The Institute of Pathology of the TU Munich provided our tissue samples. These human tissues were used after written informed consent and approval from the Ethics Review Committee of the Technical University of Munich (77/17 S). We used the tissue material which was remained after finalizing all the standard diagnostic procedures. The specimens were dissected, labeled and fixed, according to the standard procedures. All methods were carried out following the relevant guidelines.

## 5 DISCUSSION AND CONCLUSION

We demonstrated that replacing an incident Gaussian beam with a low‐order Flattened‐Gaussian beam (ie, *N* = 4) reduces the impact of secondary lobes produced by axicons significantly and improves the uniformity of the intensity profile at the central spot.

The design of the unit generating such a beam was presented. We applied this for engineering an axicon‐based static light‐sheet microscope for high‐resolution imaging.

**FIGURE 9:**
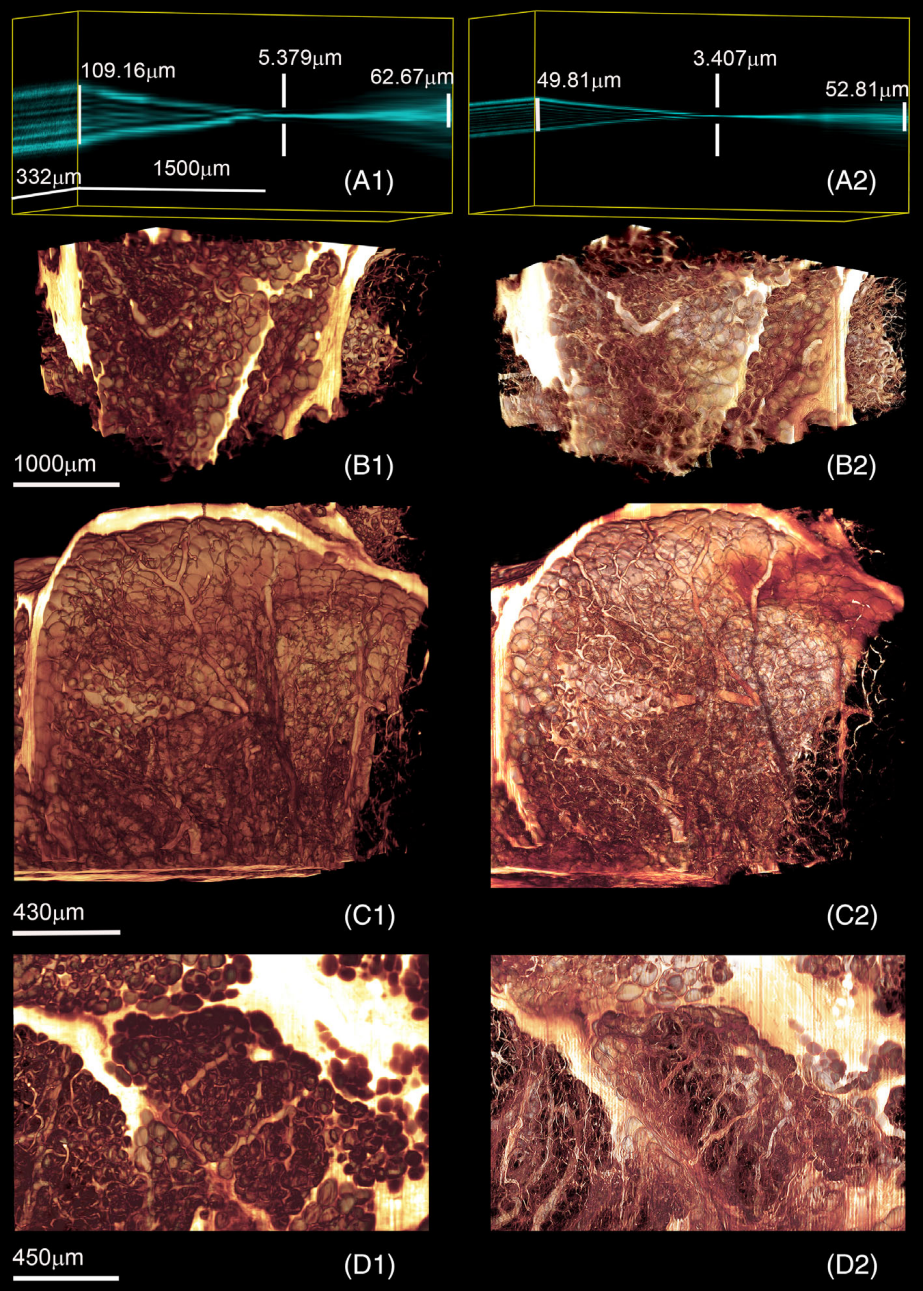
Comparing the quality of the light sheets generated by the system when the input beam has a Gaussian distribution (A1) with the ones generated by the system using an FGB of order 4 (A2). The light sheet was measured over a distance of 3000μm using a horizontal converted microscope equipped with a ×40 objective (N.A: 0.6, Olympus/Japan). The value of the beam widths presented here was obtained using Full Width Half Maximum (FWHM) method. For further comparison, the two light sheets were used for illuminating the chemically cleared human adipose mammary tissue. The images B1, C1, and D1 were recorded using an incident beam with Gaussian distribution for light sheet generation. B2, C2, and D2 show recordings of the same sections utilizing an FGB of low order (*N* = 4) as an incident beam

Figure 9A1 and A2 show a comparison between the side‐view of the light sheet produced by the system illuminated by a Gaussian beam and the light sheet produced by the system illuminated by an FGB of low order (*N* = 4) while traveling 3000 (μm) along the propagation axis.

The two different light sheets were also used for illuminating the chemically cleared human adipose mammary tissue presented in Figure 8. Comparing the images obtained by these two light sheets, a marked improvement can be observed in the images recorded by the system utilizing an FGB of low order (*N* = 4) (Figure 9B1 to D2).

To clarify the point further, the beam widths of the two light sheets are measured over 3000 μm distance at −1500 μm, −750 μm, 0 (focus), 750 μm, and 1500 μm using Full‐Width‐Half‐Maximum (FWHM) and encircling 86% of energy (D86) methods. In beam width measurements using the FWHM method, the effects of the light distributions at the tails, which have direct effects on the quality of images, are generally overlooked. These effects can be noticed with care using the D86 (1/*e* ^2^) method. The results are tabulated in Table 1. It can be concluded that the beam widths of the light sheet are improved along the propagation axis using FGB of low‐order (*N* = 4). At the focus, the beamwidth of the light sheet generated by the system using FGB of low order as input becomes smaller by a factor of 3.75 (D86)/1.57 (FWHM) compared with the light sheet generated by the same system when the incident beam has a Gaussian distribution. At +1500 μm, the beam widths of the two light sheets have almost the same value demonstrating a controlled divergence and expansion in the structure of the generated light sheet using an incident beam with FGB of low‐order distribution. Furthermore, at −1500 μm (before focus), the width of the light sheet of the system using FGB of low‐order is decreased and improved by a factor of 2.1, using either FWHM or 86% method.

**TABLE 1:** Measured beam widths of the light sheets over 3000 μm distance using full‐width half maximum (FWHM) method and encircling 86% of the energy method (D86)

Input beam profile	Measuring method	−1500 (μm)	−750 (μm)	0 (focus)	750 (μm)	1500 (μm)
Gaussian beam Input	Beam width (FWHM)	109.160 (μm)	50.55 (μm)	5.379 (μm)	11.02 (μm)	62.67 (μm)
	Beam width (86%)	125.706 (μm)	63.883 (μm)	19.862 (μm)	38.43 (μm)	148.3 (μm)
FGB (*N* = 4) Input	Beam width (FWHM)	49.81 (μm)	25.01 (μm)	3.427 (μm)	16.838 (μm)	52.81 (μm)
	Beam width (86%)	56.88 (μm)	31.35 (μm)	5.29 (μm)	47.54 (μm)	146.44 (μm)

Based on the analytical results, measured beam widths, the detected signals, and biological images, it can be seen that replacing the incident beam with a Gaussian distribution by an FGB of low‐order (*N* = 4) can create light sheets with improved optical characteristics resulting in marked improvements in final results.

As the trend in light‐sheet microscopy goes to ever‐larger samples, creating light sheets with an increased field of view is of pivotal importance. With Gaussian beams, only tiny parts of the specimen can be imaged with a subcellular and nearly isotropic resolution requiring extensive stitching. Apart from excessive recording times, this also causes strongly increased bleaching which limits the number of possible recordings that can be taken from a sample. Especially, when using staining with fluorescent dyes or antibodies bleaching becomes an important barrier that has to be considered in the imaging process.

Last but not least, apart from the significant effects on the quality of the images obtained by the static light‐sheet microscope, the described designs can be used for many other applications requiring a very thin sheet of light with high uniformity.

In the end, we want to indicate that the work presented here describes only the results that can be obtained by the use of one conic‐aspheric lens such as axicon. The combination of two or more of such elements allows additional improvements which are the subject of further work.

## Supporting information


**FIGURE S1** Top view of the axicon‐based optical unit for generating a thin sheet of light

Video S1

Video S2

Video S3

Video S4

## Data Availability

Data sharing is not applicable to this article as no new data were created or analyzed in this study.
