# Supplementary material for: Engineering a better light sheet in an axicon‐based system using a flattened Gaussian beam of low order
Source: J Biophotonics. 2022 Feb 25;15(6):e202100342. doi: 10.1002/jbio.202100342 (PMC11497253; doi:10.1002/jbio.202100342)
Supplement: Supplementary file 1 — FIGURE S1 Top view of the axicon‐based optical unit for generating a thin sheet of light [file JBIO-15-e202100342-s003.docx]

**Supplementary Material**

**Engineering a better light sheet in an Axicon-based system using a Flattened Gaussian beam of low order**

Saiedeh Saghafi *,1, Klaus Becker 1, 2, Franco Gori 3, Massih Foroughipour 1, 2, Christine Bollwein 4, Meraaj Foroughipour 1, Katja Steiger 4, Wilko Weichert 4, and Hans-Ulrich Dodt 1, 2

1 Section of Bioelectronics, Institut für Festkörperelektronik (FKE), TU Wien, Vienna, Austria

2 Section of Bioelectronics, Center for Brain Research, Medical University of Vienna, Vienna, Austria
3 Dipartimento di Ingegneria, Roma Tre University, Rome, Italy

4 Institute of Pathology, Technical University of Munich, Munich, Germany


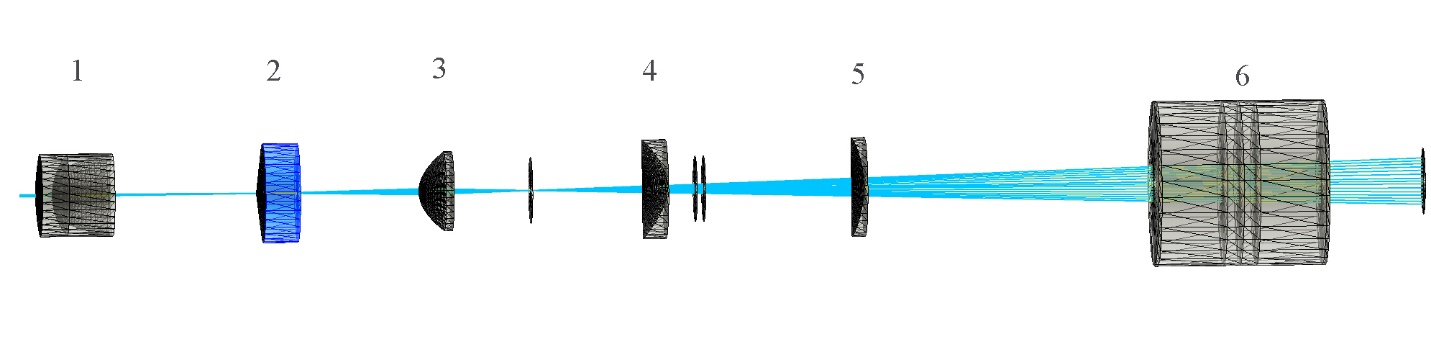
 Figure S1: Top view of the axicon-based optical unit for generating a thin sheet of light.

1. The custom-made Flattened-Gaussian-Beam-Generator-Unit (FGBGU). The FGBGU acts as an amplitude modulator. It comprises a conic aspheric surface with a radius of curvature of -7.825mm (lanthanoid family (n= 1.6591)), a 2mm apodizing soft aperture, an aspheric optical element made from Titanate (radius of curvature is 90.758mm), and a negative conic aspheric lens made from a material of the lanthanoid family with a refractive index of n=1.8685 (radius of curvature is -15.785 mm). The soft aperture has a normalized optical density that is defined by a polynomial function of order four. It is cemented to the flat surface of the first optical element.
2. An Axicon with a cone angle of 170 degrees (Asphericon/Germany)
3. A positive precision-aspherized-achromatic lens of focal length 18*mm* that is placed at 36*mm* from the plane surface of the axicon. The conic factor is -1 and the higher parameter of the aspheric structure are:

| Parameter1 | Parameter 2 | Parameter 3 | Parameter 4 |
| --- | --- | --- | --- |
| $-5.1*{10}^{-6}$ | $-1.*{10}^{-5}$ | $-1.75*10-5$ | $-9.8*{10}^{-7}$ |

1. A positive-acylinder lens of focal length 40mm (Lanthanum(S-LAH64) material) was placed at 58*mm* distance. The conic factor is -1 and the higher parameter of the aspheric structure are:

| Parameter 1 | Parameter 2 | Parameter 3 | Parameter 4 |
| --- | --- | --- | --- |
| $1.4*{10}^{-6}$ | $-1.029*{10}^{-10}$ | $-1.45*{10}^{-13}$ | $-3.07*{10}^{-17}$ |

1. A positive achromatic-cylinder doublet lens with a focal length of 75*mm* (N-Bk7 and N-SF2 materials) was placed at a distance of 56.56*mm* from the plane surface of the 40mm acylinder lens enabling us to form a very thin sheet of light.
2. A 50mm quartz specimen chamber.
